# Resilience mechanisms at work: The psychological immunity-psychological elasticity (PI-PE) model of psychological resilience

**DOI:** 10.1007/s12144-021-01813-5

**Published:** 2021-05-09

**Authors:** Richta C. IJntema, Wilmar B. Schaufeli, Yvonne D. Burger

**Affiliations:** 1 Department of Social, Health and Organizational Psychology, Faculty of Social and Behavioural Sciences, Utrecht University, P. O. box 80.140, 3508 TC Utrecht, The Netherlands; 2 Research Unit Occupational and Organizational Psychology and Professional Learning, KU Leuven, Leuven, Belgium; 3 School of Business and Economics, Center for Executive Coaching, VU University Amsterdam, Amsterdam, The Netherlands

**Keywords:** Psychological immunity-psychological elasticity (PI-PE) model, Psychological resilience, Tolerance, Narrative construction, Stressor, Adaptation

## Abstract

Recently, scientists have shifted their focus from studying psychological resilience as a single, isolated construct (e.g. attribute or outcome) to studying it as a dynamic process encompassing a number of temporally related elements. Models depicting this process explain why some people adapt to stressor exposure, whereas others do not. To date, these process models did not sufficiently explain how people adapt *differently* to a stressor. To address this issue, we developed a new model of psychological resilience, called the Psychological Immunity-Psychological Elasticity (PI-PE) model. The aim of this article is to clarify this model and to discuss its added value. First, we explain how we derived the PI-PE model from the literature regarding both the crucial elements in any resilience process model and the (mal)adaptive outcomes following stressful events. Secondly, we describe the different elements that make up the model. Characteristic of the PI-PE model is that it distinguishes between two pathways of psychological resilience – psychological immunity and psychological elasticity – with four adaptive outcomes, namely sustainability, recovery, transformation and thriving. To explain how people arrive at these different outcomes, we argue that two consecutive mechanisms are critical in these pathways: tolerance and narrative construction. Taken as a whole, the PI-PE model presents a comprehensive framework to inspire both research and practice. It explains how the process of psychological resilience works differently for different people and how to support individuals in their process towards successfully and differently adapting to stressors.

## Introduction

The intriguing question that has driven research regarding psychological resilience is why some people maintain functioning after a stressful period or event (*stressor*), whereas others do not (Crane, 2017; Fletcher & Sarkar, 2013). This question suggests that people have two options after being exposed to a stressor, either resilience or non-resilience (Van Breda, 2018). Even though this dichotomy may have an intuitive appeal, scientists nowadays agree that psychological resilience should not be treated as a single, isolated construct (e.g. outcome variable or attribute), but rather as a dynamic process by which people adapt to stressful events or circumstances they are exposed to (Bonanno et al., 2015; Fisher et al., 2019; IJntema et al., 2019). To date, research has identified several trajectories which could be considered as resilience trajectories, for example, sustainability, recovery and (posttraumatic) growth (e.g. Ayed et al., 2019; Bonanno & Diminich, 2013; Britt et al., 2016; Bryan et al., 2019; Zautra, Arewasikporn, & Davis, 2010a). *Sustainability* implies that people maintain relatively stable and healthy levels of functioning after being exposed to a stressor (Bonanno, 2004); *recovery* implies that people are negatively affected by a stressor, but are able to ‘bounce back’ (rapidly) to their pre-stressor level of functioning (Zautra, Arewasikporn, & Davis, 2010a); *growth* implies that people function better after being exposed to a stressor than before (Ayed et al., 2019).

**Fig. 1:**
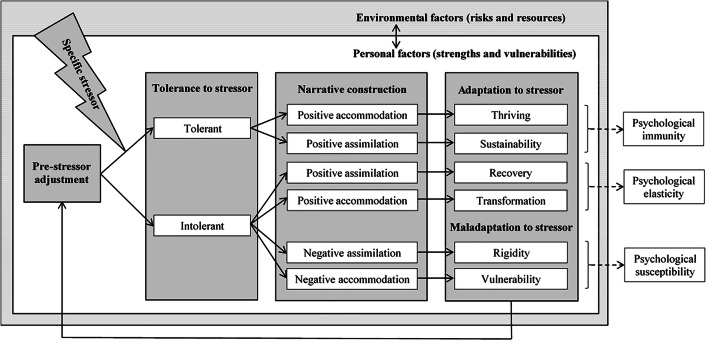
Psychological Immunity-Psychological Elasticity (PI-PE) model of psychological resilience

As there is more than one resilience trajectory, the question as to why people adapt to a stressor should be reformulated into the question as to how people adapt *differently* to a stressor. To answer this question, we need to identify which mechanisms could explain these differences. Mechanisms are the core processes or mediating variables in the resilience process (Fisher et al., 2019). Research regarding resilience mechanisms is still in its infancy. Several mechanisms have been identified in the literature, for example, stressor appraisal, seeking support, planning, coping, finding meaning and self-regulation (Britt et al., 2016; Fisher et al., 2019). However, scientists do not agree as to which mechanisms are core to the resilience process. In addition, they do not clearly distinguish mechanisms (i.e. mediating variables) from moderating variables influencing this process (IJntema et al., 2019). Finally, existing dynamic process models (e.g. Britt et al., 2016; Fisher et al., 2019; Kossek & Perrigino, 2016) do not explain by which mechanisms people adapt *differently* to stressors. To answer this question, we developed a new model of psychological resilience: the *Psychological Immunity-Psychological Elasticity (PI-PE) model* (see Fig. 1). This model introduces two consecutive mechanisms, tolerance and narrative construction, which help to explain why people arrive at different outcomes after being exposed to a specific stressor. The aim of this article is to clarify this new model (see Table 1 for an explanation of each concept in the model) and to discuss its added value.

**Table 1:** Definitions of all concepts and their function in the PI-PE model

Concept	Definition	Function in PI-PE model
Pre-stressor adjustment	The extent to which a person is psychologically adapted to a specific stressor prior to exposure to that stressor.	A setpoint for interpreting the outcome of the process of psychological resilience.
Specific stressor	A specific demanding or difficult situation a person is facing.	Stimulus that is needed to trigger the process of psychological resilience.
Tolerance to specific stressor	The extent to which a person refrains from responding defensively to a specific stressor.	The immediate response after stressor exposure and the first phase in which psychological resilience can be demonstrated.
*Tolerant*	Refraining from responding defensively to a specific stressor.	The positive extreme of the tolerance dimension.
*Intolerant*	Responding defensively to a specific stressor.	The negative extreme of the tolerance dimension.
Narrative construction	The extent to which a person is able to make sense of a stressful experience and come to terms with it.	The second phase in which psychological resilience can be demonstrated.
*Positive assimilation*	Incorporating a stressful experience into an existing narrative which is constructive for the self or the world.	A type of narrative construction.
*Positive accommodation*	Creating a new narrative which is constructive for the self or the world in order to incorporate a stressful experience.	A type of narrative construction.
*Negative assimilation*	To incorporate a stressful experience into an existing narrative which is unconstructive for the self or the world.	A type of narrative construction.
*Negative accommodation*	To construct a new narrative which is unconstructive for the self or the world in order to incorporate a stressful experience.	A type of narrative construction.
Adaptive outcomes	Successful or better-than-expected outcomes after a stressful event.	A visible manifestation of psychological resilience.
*Thriving*	Optimized psychological functioning compared to pre-stressor functioning, whereby functioning is strengthened by that stressor.	A type of adaptation.
*Sustainability*	Maintained psychological functioning by enduring a stressor and continuing forward.	A type of adaptation.
*Recovery*	Restored psychological functioning, exhibited by bouncing back (rapidly) to pre-stressor functioning (after this functioning was affected by a stressor).	A type of adaptation.
*Transformation*	Changed psychological functioning (through narrative reconstruction) compared to pre-stressor functioning (after this functioning was affected by a stressor).	A type of adaptation.
Maladaptive outcomes	Unsuccessful outcomes after a stressful event.	A visible manifestation of the absence of psychological resilience.
*Rigidity*	Restricted psychological functioning, exhibited by ineffective fixation in response to a stressor.	A type of maladaptation.
*Vulnerability*	Deteriorated psychological functioning in response to a stressor, exhibited by enhanced sensitization to that stressor.	A type of maladaptation.
Personal factors	Internal factors that influence a person’s pre-stressor adjustment, tolerance to a stressor, narrative construction and positive adaptation to a stressor.	To show that the psychological process of resilience is embedded in a specific person.
Environmental factors	External factors that influence a person’s pre-stressor adjustment, tolerance to a stressor, narrative construction and positive adaptation to a stressor.	To show that the psychological process of resilience is embedded in a specific context.
Pathway of psychological immunity	Demonstration that a person’s pre-stressor adjustment is robust enough to tolerate a specific stressor.	A pathway of psychological resilience.
Pathway of psychological elasticity	Demonstration that a person is able to construct a personal narrative that enables them to adapt to a specific stressor after their functioning was initially affected by that stressor.	A pathway of psychological resilience.
Pathway of psychological susceptibility	Demonstration that a person is not able to construct a personal narrative that enables them to neither be immune nor adapt to a specific stressor after their functioning was initially affected by that stressor.	A maladaptive pathway.

## Psychological Immunity-Psychological Elasticity Model

The PI-PE model is restricted to *psychological* resilience, which emphasizes resilience in psychological functioning: the interplay between a person’s behaviour, cognition and affect at a certain time in a certain context (IJntema et al., 2019). We derived this model from the existing literature regarding the crucial elements in any process model of psychological resilience (Bonanno et al., 2015; Fisher et al., 2019; IJntema et al., 2019) and from the literature regarding adaptive and maladaptive outcomes following stressful events or circumstances (e.g. Ayed et al., 2019; Carver, 1998; Zautra, Arewasikporn, & Davis, 2010a). As can be seen in Fig. 1, the PI-PE model consists of two consecutive mechanisms, three conditions and the outcome of (mal)adaptation. The two mechanisms are *tolerance* to a specific stressor and *narrative construction*. The three conditions are 1) *pre-stressor adjustment*, which functions as a reference point to determine whether adaptation to a stressor has occurred; 2) the *stressor*^1^ as the critical condition to trigger the process of resilience; and 3) *personal and environmental factors* as the moderating variables that influence the relationship between the stressor, resilience mechanisms and resilience outcomes. The outcome of *(mal)adaptation* to the stressor is the visible manifestation of the (lack of) resilience. The PI-PE model distinguishes four adaptive outcomes – *sustainability*, *recovery*, *transformation* and *thriving* (e.g. Bonanno, 2004; Carver, 1998; Tedeschi & Calhoun, 2004; Zautra, Arewasikporn, & Davis, 2010a) – and two maladaptive outcomes – *rigidity* and *vulnerability* (e.g. Friborg et al., 2009; Niesen et al., 2014). In this article, we explain in more detail these elements that make up the PI-PE model.

The unique contribution of the PI-PE model are the resilience mechanisms of tolerance and narrative construction. By including these two mechanisms, three pathways emerge that illustrate how people arrive at different (mal)adaptive outcomes after being exposed to the same stressor: 1) the pathway of *psychological immunity* resulting in the adaptive outcomes of either sustainability or thriving; 2) the pathway of *psychological elasticity* resulting in the adaptive outcomes of either recovery or transformation; and 3) the pathway of *psychological susceptibility* resulting in the maladaptive outcomes of either rigidity or vulnerability. We consider the first two pathways as resilient or adaptive pathways and the third pathway as a non-resilient or maladaptive pathway. A special feature of the model is the arrow from (mal)adaptation back to pre-stressor adjustment in Fig. 1. This arrow illustrates that the PI-PE model repeats each time a person encounters a (similar) stressor as well as that people can learn from their experience with a specific stressor.

In this article, we define psychological resilience as a dynamic process by which people adapt to a specific stressor. This process is triggered by a specific stressful event/circumstance and is aimed at enhancing, maintaining, restoring or altering psychological functioning, either via the pathway of psychological immunity or via the pathway of psychological elasticity. Below, we explain in corresponding sections all the elements depicted in the PI-PE model (see Fig. 1): pre-stressor adjustment, specific stressor, tolerance, narrative construction, (mal)adaptive outcomes, and personal and environmental factors. Subsequently, we explain the pathways of psychological immunity, psychological elasticity and psychological susceptibility. In the discussion, we reflect on the added value of the PI-PE model, its applicability, its limitations and its relevance for future research and practice.

### Pre-Stressor Adjustment

Many process models of psychological resilience start with a stressor (e.g. Britt et al., 2016; Kossek & Perrigino, 2016; McLarnon & Rothstein, 2013). However, it is hard to determine whether positive adaptation to a specific stressor has occurred – the outcome of the process of psychological resilience – without having information about the extent to which a person was adjusted to that stressor prior to exposure (Bonanno et al., 2015; IJntema et al., 2019). That is why the PI-PE model starts with a person’s pre-stressor adjustment. We define pre-stressor adjustment as the extent to which a person is psychologically adapted to a stressor prior to being exposed to that stressor (see Table 1). It functions as a setpoint for interpreting the outcome of the process of psychological resilience (Bonanno et al., 2015). This kind of adjustment may be the consequence of previous experience(s) with that specific stressor. For example, a person facing job loss may have had experience with job loss in the past. This previous experience (either positive or negative) will influence the new experience with job loss. It is not always possible to collect data on a person’s pre-stressor adjustment, especially when the moment of stressor occurrence is unpredictable (Bonanno et al., 2015).

### Specific Stressor

A stressor is regarded as the antecedent or stimulus that is needed to trigger the process of psychological resilience (Fletcher & Sarkar, 2013; Windle, 2011). Without a stressor, psychological resilience will not emerge (Bonanno et al., 2015). Therefore, this crucial element is included in the PI-PE model. We define a stressor as a specific demanding or difficult situation a person is facing (similar to Seyles definition of a stressor as an environmental demand, see Lazarus & Folkman, 1984; see Table 1). Stressors may come in different shapes and sizes. For example, a stressor may be challenging (e.g. skill demands) or hindering (e.g. role conflict; Crane & Searle, 2016); time-bound/acute (e.g. job rejection) or ongoing (e.g. being bullied; Bonanno et al., 2015); mild (e.g. daily hassles at work, such as having an argument with a colleague) or strong (e.g. job loss; Fletcher & Sarkar, 2013); sudden (e.g. an accident) or expected (e.g. negative review after underperformance; Zautra, Hall, & Murray, 2010b); infrequent or frequent (e.g. uncivil treatment by customers; Fisher et al., 2019). In short, a stressor could be any demanding or difficult situation a person is facing, whereby the characteristics of the stressor (nature, duration, intensity, predictability and frequency; Britt et al., 2016) will influence the process of psychological resilience.

The PI-PE model assumes that psychological resilience does not emerge after stressors in general, but only in relation to a *specific* stressor. In the case of multiple or cumulative stressors, the PI-PE model assumes that psychological resilience may only develop in relation to one specific stressor at a time. Any other stressor is regarded as an environmental factor. For example, by successfully overcoming bankruptcy, an entrepreneur demonstrates psychological resilience to that specific stressor and not automatically to other stressors as well. If the bankruptcy creates marital problems, these problems function as an environmental factor that may hinder successful adaptation to bankruptcy. And if the entrepreneur demonstrates psychological resilience to bankruptcy, this does not automatically mean that they will demonstrate resilience to marital problems as well.

### Tolerance

The first mechanism in the PI-PE model is tolerance to a specific stressor. We noticed that the term ‘tolerance’ is often used in the (occupational) resilience literature (e.g. Davydov et al., 2010; Kossek & Perrigino, 2016), but not included in any process model of psychological resilience. We define tolerance as the extent to which a person refrains from responding defensively to a specific stressor (see Table 1; comparable to *stress tolerance*, see Izutsu et al., 2004). This definition implies that we consider tolerance on a continuum. We merely added the dichotomy between tolerant and intolerant behaviour in the PI-PE model (see Fig. 1) to clarify the different pathways that people may take after stressor exposure. We consider tolerance to be a critical mechanism in the dynamic process of psychological resilience because it explains why some people are not affected by a specific stressor and maintain functioning, whereas others do not. Those that maintain functioning demonstrate that their pre-stressor adjustment is robust enough to accept and endure the specific stressor. Therefore, we included tolerance to a specific stressor in the PI-PE model as the immediate response to that stressor and the first phase in which psychological resilience could be demonstrated.

We conceptualize tolerance as actual behaviour (*applied tolerance*) and not as merely accepting something one does not like (Van Doorn, 2016). Typically, tolerance includes a paradox (Van Doorn, 2014): despite the presence of a stressor, a tolerant person refrains from a stress response. For example, an employee who ‘tolerates’ a reorganization in the company, may cooperate but at the same time may not be in favour of that reorganization. A more natural reaction to a stressor would be the opposite ‘intolerance’: a defensive or stress response, to fight, flight or freeze (Woolfolk et al., 2007). An employee who does not tolerate the reorganization may call in sick more often or display counterproductive behaviour. As such, they may act more in line with opposing thoughts and negative emotions about the reorganization. Because of its paradoxical nature, tolerance is considered to be a learned response and intolerance – or the stress response – a natural response (Van Doorn, 2016).

Since tolerance depends on a specific stressor, a person can be tolerant to one stressor, but not to another. Tolerance to a specific stressor may be acquired by successfully dealing with that stressor. Those mastery experiences strengthen a person’s resistance to similar future stressors. This process is referred to as the *steeling effect* of adversity (Rutter, 1985, 2012). The person is better prepared for adversity in the future (*psychological preparedness*; Janoff-Bulman, 2004). The opposite could also happen, the *sensitizing effect* of adversity (Rutter, 1985, 2012). In this case, the stressful experience does not strengthen the person, but makes the person more susceptible to similar future stressors. Instead of tolerance, the person has acquired intolerance to the particular stressor. In the case of an ongoing stressor, people may be tolerant up to a certain level or threshold, at which their tolerance turns into intolerance.

### Narrative Construction

The second mechanism in the PI-PE model is narrative construction. We adopted this term from Meichenbaum (2006), who emphasized the critical role of the self-narrative for resilience. We define narrative construction as the extent to which a person is able to make sense of their experience and come to terms with it (Wilson, 2011; see Table 1). We consider it as a critical mechanism in the dynamic process of psychological resilience because it explains why some people, once affected by a stressor, are able to bounce back and others do not. Those that bounce back demonstrate that they have the personal and environmental resources to make sense of the experience and come to terms with it. If psychological resilience was not demonstrated in the first phase because of an intolerant response, narrative construction signifies the second phase in which psychological resilience could be demonstrated.

Narrative construction may require more or less effort depending on the impact of the stressor on a person’s basic assumptions, which are ‘beliefs that ground, secure, or orient people, that give a sense of reality, meaning or a purpose in life’ (Kauffman, 2002, p. 1). Highly stressful or traumatic events are known to have a disruptive effect on a person’s basic assumptions (Janoff-Bulman, 1992; Joseph & Linley, 2005; Parkes, 1971; Tedeschi & Calhoun, 2004). The ‘shattered assumptions theory’ states that traumatic events disrupt beliefs related to the benevolence and meaningfulness of the world and the worthiness of the self (Janoff-Bulman, 1992). Scientists refer to these core assumptions in terms such as *worldview*, *higher-order schemata* (Calhoun & Tedeschi, 1999), *assumptive world* (Janoff-Bulman, 2004; Parkes, 1971), *self-narrative* (Neimeyer, 2006) and *core narrative* (Wilson, 2011). When disruption occurs there is a need to revise, repair or replace basic assumptions to integrate new information (Joseph & Linley, 2005; Neimeyer, 2006). As such, narrative construction is a way of coping with stressors (Neimeyer & Levitt, 2001).

As can be seen in Fig. 1, we subdivided narrative construction into positive accommodation/assimilation and negative accommodation/assimilation. We based this distinction on the ‘organismic valuing theory of growth through adversity’ (Joseph, 2009; Joseph & Linley, 2005). This theory has adopted the Piagetian concepts of *accommodation* and *assimilation* to distinguish between two different processes in narrative construction. When the stressor does not have a disruptive effect on people’s core narratives, they will be able to assimilate the experience within existing narratives about themselves and the social world. As such, assimilation does not require a change in core narratives, but ‘only’ a change in the interpretation or meaning of the event to make it less contradictive to their core narratives. However, when the stressor has a disruptive effect on people’s core narratives, assimilation is not possible. In that case, people need to change their existing narrative and construct a new narrative about themselves and/or the world in accord with lessons learned from the experience. We also make a distinction between *positive* and *negative* assimilation/accommodation. Positive assimilation/accommodation is directed at growth and leads to adaptation. Negative assimilation/accommodation is distress-focused and leads to maladaptation (for more information on assimilation and accommodation, see Brandtstädter & Rothermund, 2002; Leipold & Greve, 2009). Note that we subdivided narrative construction in the PI-PE model merely to clarify the different pathways that people may take after stressor exposure, rather than to draw a hard line between these subdivisions.

### (mal)Adaptative Outcomes

The outcome of the process of psychological resilience should be some type of positive adaptation, because only a successful or better-than-expected outcome after a stressful event implies resilience (Van Breda, 2018; Windle, 2011). Positive adaptation is regarded as the visible manifestation of psychological resilience (Fisher et al., 2019). Therefore, we included it as a crucial element in the PI-PE model. The term ‘positive’ denotes a type of adaptation that leads to an enhanced sense of mastery (Earvolino-Ramirez, 2007). In the introduction of this article, we distinguished three types of positive adaptation: sustainability, recovery and growth. As can be seen in Fig. 1, the PI-PE model does not include (posttraumatic) growth, but rather thriving and transformation, which is a more specific distinction that has been made in the literature (Carver, 1998; Lepore & Revenson, 2006; Ryff & Singer, 2003). Below, we discuss these four types of positive adaptation and contrast them with two types of maladaptation, that are indicative of poor psychological resilience: rigidity and vulnerability.

#### Sustainability

Sustainability, also known as *resistance* (Lepore & Revenson, 2006) or *robust resilience* (Fletcher & Sarkar, 2016), is a type of positive adaptation that we define as maintained psychological functioning by enduring a stressor and continuing forward (see Table 1; based on the definition of Zautra, 2009). It emphasizes the ability of a person to maintain a stable equilibrium of healthy functioning in the face of a stressor (Ayed et al., 2019; Bonanno, 2004). The difference with non-resilience is, that a resilient person is relatively unaffected by the stressor and able to continue to function capably (tolerant), whereas a non-resilient person is affected by the stressor and experiences a period of malfunctioning (intolerant).

#### Recovery

Recovery or *rebound resilience* (Fletcher & Sarkar, 2016) is a type of positive adaptation that we define as restored psychological functioning, exhibited by bouncing back (rapidly) to pre-stressor functioning after this functioning was affected by a stressor (based on the definition of Smith et al., 2010; see Table 1). Recovery assumes that people experience a period of distress or malfunctioning after being exposed to a stressor and are able to return to previous pre-stressor levels of functioning (Ayed et al., 2019). This is known as the principle ‘homeostasis’ (a term coined by Cannon in the 1920s): a return to a former, more balanced state. In the resilience literature, recovery is not restricted to the recovery process itself, but is used for comparing a person’s recovery to what is considered a ‘normal’ standard of recovery (Zautra, Hall, & Murray, 2010b). Thus, a resilient person recovers more quickly than a less resilient person (Martin-Breen & Anderies, 2011). Environmental and personal factors may hinder or support the extent and speed of recovery (Zautra, Hall, & Murray, 2010b).

#### Transformation

Transformation or *reconfiguratio*n (Lepore & Revenson, 2006) is a type of positive adaptation that we define as changed psychological functioning (through narrative reconstruction) compared to pre-stressor functioning after this functioning was affected by a stressor (see Table 1). In the case of highly stressful events, such as a life threatening disease, the loss of a loved one or combat, transformation is similar to posttraumatic growth (PTG), which is defined as ‘the experience of positive change that occurs as a result of the struggle with highly challenging life crises’ (Tedeschi & Calhoun, 2004, p. 1). Transformation emphasizes the life-changing effect of struggling with a stressful event (Tedeschi & Calhoun, 2004). Similar to recovery, the person’s functioning is affected by the stressor (intolerant). In contrast to recovery, the person is unable to incorporate the experience into an existing frame of mind (assimilation). Instead, the person has to change an existing frame of mind in order to integrate the stressful or traumatic experience (accommodation; Lepore & Revenson, 2006). The stressor symbolizes a turning point (Rutter, 1999) or a transition (Parkes, 1971) in a person’s life. A resilient person is able to make that turn in life, to make sense of the experience and to come to terms with it, whereas a non-resilient person is not.

#### Thriving

Thriving differs in one important aspect from the other three types of positive adaptation: a stressor is not a necessary condition for thriving. This is reflected in the definition of thriving: ‘a psychological state in which individuals experience both a sense of vitality and a sense of learning’ (Spreitzer et al., 2005, p. 538). We confine ourselves to thriving in the face of a stressor and define it as optimized psychological functioning compared to pre-stressor functioning, whereby functioning is strengthened by that stressor (see Table 1). In this sense, thriving emphasizes the benefits that may be associated with passing through a challenging experience (Carver, 1998). These benefits can be a new skill, self-knowledge, confidence and strengthened personal relationships or resources (Carver, 1998; Ryff, 2014). Thriving and sustainability have in common that a person’s functioning is not negatively affected by the stressor (tolerant), in contrast to recovery and transformation where the person’s functioning is negatively affected by the stressor (intolerant). Recovery and sustainability do not require a person to change their frame of mind (assimilation), while thriving and transformation do require a change in frame of mind (accommodation).

#### Rigidity

People do not always adapt after being exposed to a stressor. The exposure can also lead to maladaptation and one particular maladaptive outcome is rigidity. Psychological rigidity has been defined as persistence in a course of action that is possibly no longer the best way to solve the problem or to reduce the threat (Cowen, 1952; Niesen et al., 2014). We define rigidity as restricted psychological functioning, exhibited by ineffective fixation in response to a stressor (see Table 1). According to the threat-rigidity thesis (Staw et al., 1981), adverse events evoke anxiety and stress in people which narrow down perception and information processing (*restriction of information*) and restrict their behavioural repertoire in responding appropriately to stressors (*constriction of control*). The displayed behaviour is less varied or flexible, more habitual and more fixed or rigid. The threat-rigidity thesis emphasizes that people often react to stressors with well-learned behaviours and habitual responses. If a habitual response is ineffective to adapt to a stressor, we regard it as rigidity. However, if the habitual response is effective, we regard it as sustainability. The similarity between rigidity and sustainability is that both result in no fundamental change in psychological functioning (assimilation). The difference is that assimilation is negative (ineffective) in the case of rigidity and positive (effective) in the case of sustainability. In essence, rigidity is maladaptive as the context demands a different reaction from the person to adapt, namely to positively accommodate the experience, rather that assimilate it.

#### Vulnerability

Vulnerability is by some authors conceived of as the negative counterpart of resilience (Friborg et al., 2009; Kaplan, 2013). In this article, we do not adopt a single dimension perspective as we distinguish four adaptive and two maladaptive outcomes. We consider vulnerability as one type of maladaptation and define it as deteriorated psychological functioning in response to a stressor, exhibited by enhanced sensitization to that stressor (see Table 1). A person is more fragile after the stressor than before (London, 1983). Vulnerability is comparable to ‘chronic dysfunction’ (Bonanno et al., 2015; Bonanno & Diminich, 2013). Both vulnerability and transformation are characterized by intolerance to the perceived stressor and by the accommodation of an existing frame of mind to incorporate the adverse experience. However, the difference is that transformation is associated with positive (effective) accommodation, whereas vulnerability is associated with negative (ineffective) accommodation. The latter causes the person to be more sensitive to a future stressor than before stressor exposure (*negative cascading effect*; Masten, 2014).

### Personal and Environmental Factors

The PI-PE model describes the psychological process a person goes through after being exposed to a stressor. This psychological process does not occur in isolation, but is embedded in a specific person and in a specific context. As such, both personal and environmental factors are influencing this psychological process. We define personal and environmental factors as internal and external influences on a person’s pre-stressor adjustment, tolerance, and narrative construction and on (mal)adaptive outcomes (see Table 1). *Personal factors* are internal strengths and vulnerabilities, such as (a lack of) self-efficacy, optimism, motivation, hope and perspective (Ayed et al., 2019; Bryan et al., 2019). In a meta-analytic study, self-efficacy, positive affect and self-esteem were found to be the strongest personal protective factors against stressors (Lee et al., 2013). In addition, we suggest to consider control, commitment and challenge as personal factors, as they are all components of the resilience-related concepts of hardiness (Kobasa, 1979; Maddi, 2002) and mental toughness (Clough et al., 2002). *Environmental factors* are external risks and resources, such as other stressors and (a lack of) support. The support can come from close relationships (partner, family, friends) as well as social networks (related to work, sport, leisure, common interest or religion) and the community (e.g. neighbourhood, school, work, village/town, virtual, national, international; Ayed et al., 2019; Bryan et al., 2019; Masten, 2014; Windle, 2011). Research has identified many personal and environmental factors (see, for example, Britt et al., 2016; Bryan et al., 2019; Fisher et al., 2019), also known as ‘protective factors’, ‘promoting factors’ and ‘adaptive factors’ (Davydov et al., 2010; Fletcher & Sarkar, 2013; Masten, 2014).

In the resilience literature, the question has been raised to what extent personal and environmental factors, such as self-efficacy and social support, are different from factors that are associated with good health and development in general (Masten, 2014). These general health factors are also referred to as *general adaptive systems* that protect humans under many different circumstances (Masten, 2014) and as *general resistance resources* that facilitate successful coping with the inherent stressors of human existence *(salutogenesis*; Antonovsky, 1996). According to Fisher et al. (2019), a ‘defining feature of the variables in this category is that they are present irrespective of whether someone experiences adversity, but nonetheless can provide a protective or ameliorative function in the event that adversity does occur’ (p. 11). Therefore, we do not consider the personal and environmental factors in the PI-PE model to be different from health factors in general.

### Three Pathways

The mechanisms of tolerance and narrative construction are central to the three pathways depicted in the PI-PE model: 1) psychological immunity, 2) psychological elasticity and 3) psychological susceptibility. The first two pathways are emphasized in the title of the PI-PE model as they imply resilience. The mechanism that distinguishes between these two pathways is tolerance. The third pathway is included in the PI-PE model by way of contrast as it illustrates the absence of resilience. The mechanism that distinguishes this pathway from the other two pathways is narrative construction. The first two pathways are named after two commonly used metaphors for psychological resilience: *psychological immunity* (Davydov et al., 2010; Shastri, 2013) and *elasticity* (e.g. a spring, elastic band, elasticity of metal or a bending tree; Fletcher & Sarkar, 2013; Masten, 2014). Psychological immunity is a pathway in which a person demonstrates that their pre-stressor adjustment is robust enough to tolerate a specific stressor. This pathway results in the adaptive outcome of either sustainability or thriving, depending on narrative construction. Psychological elasticity is a pathway in which a person – who’s functioning was initially affected by the stressor (intolerant) – is able to bounce back and adapt to the stressor. This pathway results in the adaptive outcome of either recovery or transformation. To the best of our knowledge, the PI-PE model is the first model to combine these two metaphors into one model.

For most people, the pathway of psychological immunity will be most appealing. In this pathway, a person is able to endure the event (tolerance), manage the event (availability and use of personal and environmental factors), and make sense of the experience and find closure (positive assimilation or accommodation). The pathway of psychological elasticity is more demanding because a person must at least deal with the negative effects of being intolerant to a specific stressor and – in the case of transformation – also construct a new narrative or alter an existing narrative about the self and the social world to adapt to that stressor. This dual process leading to transformation in the PI-PE model resembles the dual process in coping with bereavement: the processing of an experience of loss and the struggle to reorient oneself in a changed world (Stroebe & Schut, 2010).

Which pathway a person might be involved in is not by choice, but depends on the two mechanisms and three conditions: 1) the person’s pre-stressor adjustment; 2) the nature, duration and intensity of the specific stressor; 3) their tolerance to that specific stressor; 4) the extent to which this person is able to integrate their experience into an existing narrative (assimilation) or in a new or altered narrative about the self and the social world (accommodation); and on 5) the availability and use of personal and environmental resources to deal with that stressor. Whatever the outcome, this experience will become part of a person’s psychological functioning and thus pre-stressor adjustment for similar stressors in the future. This is illustrated by the arrow from (mal)adaptation back to pre-stressor adjustment in Fig. 1. An experience with a specific stressor may either help a person to learn to tolerate a similar stressor in the future (*upward spiral*) or it may cause a person to become more intolerant to that stressor (*downward spiral*). As people constantly face new stressors and many stressors recur, the process of psychological resilience is a continuous, recurring process.

## Discussion

The aim of this article was to answer the question by which mechanism people adapt differently to a stressor. To answer this question, we developed a new dynamic process model of psychological resilience: the Psychological Immunity-Psychological Elasticity (PI-PE) model (see Fig. 1). This model clarifies the different pathways that people may follow after they encountered a specific stressor. The first pathway is psychological immunity which results in either enhanced psychological functioning (thriving) or maintained psychological functioning (sustainability), compared to pre-stressor functioning. The second pathway is psychological elasticity which results in either restored psychological functioning (recovery) or altered psychological functioning (transformation). The third pathway is psychological susceptibility which results in either restricted psychological functioning (rigidity) or deteriorated psychological functioning (vulnerability). The PI-PE model shows that two mechanisms explain which pathway people take: 1) tolerance to the stressor explains whether people take the first or second pathway after stressor exposure; 2) narrative construction explains whether people take the second or the third pathway. To answer our research question, people adapt differently to a specific stressor because they differ with respect to their tolerance to that stressor, and the personal narrative they construct to make sense of their stressor experience and find closure.

A strength of the PI-PE model is that it not only explains how people adapt differently to a stressor, but also explains why the resilience process works differently for different people. The three conditions in the model help explain these differences: people’s history and experience with the stressor (pre-stressor adjustment); the nature, duration and intensity of the specific stressor they are facing; and the availability and use of their personal and environmental resources (see Table 1 for the meaning of these different elements). Hence, the resilience process works differently for different people, because people differ with respect to these three conditions.

Another strength of the PI-PE model is that it explains how the same person can respond differently over time to the same stressor. This is because the resilience process repeats each time a person encounters a stressor and people can learn from their experience with a specific stressor. When encountering a specific stressor for the first time, the PI-PE model assumes that it is unlikely that people respond by demonstrating tolerance because intolerance is considered as the ‘default’ response (Van Doorn, 2016). However, tolerance can be acquired over time by successfully dealing with that stressor. The experience will become part of a person’s psychological functioning and thus pre-stressor adjustment for similar stressors in the future. As stressors are part of daily (work)life, it is very likely that people have learned to adapt to many stressors in their lives already, often without even realizing it. However, it could also be that people have developed maladaptive narratives to cope with certain stressors (Young et al., 2003) as a consequence of having to deal with ‘toxic’ adverse childhood experiences (ACEs; Felitti et al., 1998), such as abuse.

The added value of the PI-PE model lies in that it synthesizes the elements that are considered ‘standard’ elements in dynamic process models of psychological resilience: pre-stressor adjustment, a stressor, resilience mechanisms, resilience resources and resilience outcomes (IJntema et al., 2019). In addition, the model includes the outcomes that have been related to resilience before: sustainability, recovery, transformation and thriving (Carver, 1998; Tedeschi & Calhoun, 2004; Zautra et al., 2010b). By doing so, the PI-PE model establishes a link between research regarding psychological resilience (e.g. Bonanno et al., 2015; Masten, 2014), regarding coping under stress (e.g. Lazarus & Folkman, 1984), and regarding posttraumatic growth (e.g. Tedeschi & Calhoun, 2004). These three research domains focus on how people adapt to adverse and stressful circumstances. The main difference is, that research regarding resilience and posttraumatic growth – by definition – focuses on positive outcomes after stressor exposure, while the outcome in stress-coping research may be either positive or negative (Fletcher & Sarkar, 2013). Resilience and posttraumatic growth research differ with respect to the type of positive outcome: posttraumatic growth research focuses on growth or transformation after stressor exposure, while resilience research is more focused on maintaining or recovering to normal daily functioning (Levine et al., 2009; Zautra, Hall, & Murray, 2010b). By introducing the concepts of tolerance and narrative construction, the PI-PE model not only combines the different (mal)adaptive outcomes into one model, but also clarifies by which mechanisms people arrive at different (mal)adaptive outcomes after stressor exposure.

The implication of taking a process-based perspective on psychological resilience is that the factor ‘time’ needs be taken into account (Britt et al., 2016; Fisher et al., 2019). First, the timescale of the stressor should be taken into account as stressors differ in duration and may be either time-bound or chronic. In addition, the timescale of the process of resilience itself should be taken into account as narrative construction may take more or less time. To illustrate, in the case of an acute stressor (e.g. job rejection), a person may have an immediate stress response (intolerant) to that stressor, but transforms quickly by reappraising the situation (narrative construction). In the case of a chronic stressor (e.g. workload), a person might show tolerance to this stressor in the first weeks and sustain functioning. However, if the stressor continues to be present for months a person’s tolerance could turn into intolerance if they are not able to accommodate their narrative or lack the personal or environmental resources to deal with the chronic stressor.

At first sight the PI-PE model may be understood as a cognitive-behavioural model as it makes a clear distinction between a person’s capacity for and demonstration of resilience (a distinction emphasized by Britt et al., 2016). In the pathway of psychological immunity, pre-stressor adjustment is considered as the capacity for resilience and tolerance to a stressor as the demonstration of resilience. This implies that people are resilient as their pre-stressor adjustment is robust enough to tolerate a specific stressor in a way that is visible to others. In the pathway of psychological elasticity, narrative construction is considered as the capacity for resilience and positive adaptation as the demonstration of resilience. This implies that people are resilient if they are able to construct a personal narrative that enables them to adapt to a stressor in a way that is visible to others. However, to conceive the PE-PI model – based on this distinction – as a cognitive-behavioural model is too simple. After all, the PI-PE model does not regard a person’s psychological process in isolation, but acknowledges that the whole process is embedded in a specific person-environment interaction. Therefore, we regard the PI-PE model as a *biopsycho-ecological* model.

The PI-PE model is broadly applicable: everyone faces stressors in their (work) life and the model describes the psychological process that people go through after being exposed to a stressor. An important condition for applying the model is that a *specific* stressor can be identified as the PI-PE model assumes that psychological resilience can only be acquired for specific stressors, not to stressors in general.

### Limitations

In this article, we make a strong argument for the PI-PE model. It is grounded in a large body of research regarding resilience, stress and post-traumatic growth and is best understood as a comprehensive framework for understanding how the process of psychological resilience works differently for different people over time. Yet, the model is not without limitations. The first limitation is that the PI-PE model focuses on the psychological process of resilience and not on the physical or physiological process. However some stressors affect a person both psychologically and physically, for example, natural disasters and accidents (external stressors) or a physical illness (internal stressor; Stewart & Yuen, 2011). In these instances, the PI-PE model focuses on the psychological process of adapting to the encountered stressor and considers the physical process as a moderating factor influencing this psychological process.

The second limitation of the PI-PE model is that it is restricted to specific stressors and that it is not applicable to stressors in general. Note, that this implies that the PI-PE model is not fit for crisis situations, such as the current COVID-19 crisis, where many stressors coincide. When a person encounters several stressors at the same time, different resilience processes get in motion. For example, a person could be facing a negative job review at work and the loss of a parent a home. Both resilience processes should be treated separately as each process may have a different outcome. At the same time, each process influences the other. The loss of a parent should be considered as an environmental factor influencing the adaptation to the negative job review and vice versa. Therefore, in the application of the PI-PE model it is very important to be explicit which stressor a person is facing and which other stressor(s) may possibly influence this process as an environmental factor.

The third limitation of the PI-PE model is that it may be hard to test the model as a whole. We consider three challenges in testing the model. First, it may be hard to isolate one resilience process from another as people are often involved in several resilience processes at the same time. Secondly, in the case of unpredictable stressors, it may be hard to capture the onset of the process which is before stressor exposure. Thirdly, it may be hard to determine when the resilience process is over as adaptation depends on both the speed of narrative construction and the availability and us of personal and environmental resources. Given these challenges, it is best to either test the PE-PE model longitudinally or investigate just parts of the model (see nest section).

### Recommendations for Future Research

Research has identified many resilience mechanisms that could help explain why people positively adapt to stressors in their (work)life (Fisher et al., 2019). Up until now, research has not answered the question by which mechanisms people arrive at different outcomes after being exposed to a stressor. The PI-PE model is the first model that answers this question by introducing two mechanisms: tolerance and narrative construction. Future research regarding the dynamic process of psychological resilience should take these two mechanisms into consideration. To learn more about tolerance, differences could be investigated between employees who demonstrate tolerance to a specific stressor and employees who do not: to what extent do these groups differ with respect to their experience with that stressor? In addition, future research could study employees who have no experience with a specific stressor: how do they respond to a new stressor and to what extent is intolerance their ‘default’ response? To learn more about narrative construction, future research could study people who have (a lot of) experience with a specific stressor: which narratives have they constructed around that stressor and to what extent do these narratives relate to (different) adaptations to that stressor? Answering these research questions would not only provide empirical evidence for the PI-PE model in itself, but also provide new insights as to how people adapt differently to stressors over time.

### Recommendations for Practice

Up until now, research has shown that adult resilience-building programmes vary considerably when it comes to the programme approach (e.g. Leppin et al., 2014; Macedo et al., 2014; Robertson et al., 2015). The PI-PE model contributes to more clarity about the best approach to enhance resilience to a specific stressor. From the PI-PE model, two main approaches can be derived. The first is a *tolerance-enhancement approach* which is a proactive approach aimed at enhancing tolerance to a specific stressor before stressor exposure. This approach is preferably applied in the case of an unavoidable stressor, which refers to a stressor that particular (groups of) people face on a regular basis and that cannot be prevented (Card, 2018), for example, teachers facing noncompliant behaviour of students and palliative care providers dealing with the death of a patient. To function effectively, people need to learn to tolerate these stressor over the longer term. Programmes adopting a tolerance-enhancement approach may exist under different names, such as ‘stress inoculation’ or ‘stress exposure training’ (e.g. Meichenbaum, 1985) and ‘(emergency) preparedness training’ (e.g. Qureshi et al., 2002). The second approach is a *narrative approach* which is a reactive approach aimed at helping people to construct narratives that help them to adapt to a specific stressor during or after stressor exposure. Programmes using this approach may exist under different names as well, such as a ‘cognitive-behavioural programme’ (e.g. Robertson et al., 2015), as ‘debriefing’ (e.g. Adler et al., 2011) or as ‘critical incident stress debriefing’ (e.g. Malcolm et al., 2005; Mitchell et al., 2003).

In addition to these two main approaches, three other approaches to resilience building can be derived from the PI-PE model. The first is a *personal resource-based approach* aimed at enhancing internal resources, such as self-efficacy. This personal approach is quite common when it comes to resilience building (see Vanhove et al., 2016). The second is an *environmental resource-based approach* aimed at enhancing external resources, such as social support. This approach is strongly advocated by Ungar (2018). Both resource-based approaches are generally applicable throughout the resilience process: before, during and after stressor exposure. The third is a *measured approach* which aims to reduce the intensity and duration of the stressor before or during stressor exposure. Since the presence of a stressor is a necessary condition for resilience, removing the stressor is not a resilience-building approach. In sum, five approaches to resilience-building can be derived from the PI-PE model: 1) a tolerance-enhancement approach; 2) a narrative approach; 3) a personal resource-based approach; 4) an environmental resource-based approach; and 5) a measured approach. These approaches contribute to more clarity about ways to support people in building their psychological resilience to a specific stressor.

## Data Availability

This manuscript has no associated data.

## Footnotes

1 We do not use the term ‘adversity’, that is often used in the resilience literature, because it is associated with events that lead to negative outcomes. Instead, we prefer to use the term ‘stressor’, because it is associated with demanding events that may result in both negative and positive, ‘resilient’ outcomes (Fletcher & Sarkar, 2013).
